# Electrotherapy Plus Photobiostimulation for the Treatment of Mild Pelvic Organ Prolapse and Stress Urinary Incontinence

**DOI:** 10.3390/jcm15052016

**Published:** 2026-03-06

**Authors:** Teresa Maria Manni, Desirèe De Vicari, Mariachiara Palucci, Alice Cola, Marta Barba, Matteo Frigerio

**Affiliations:** Department of Obstetrics and Gynecology, ASST Monza, San Gerardo dei Tintori Hospital, University Milano-Bicocca, 20900 Monza, Italy; d.devicari@campus.unimib.it (D.D.V.); m.palucci1@campus.unimib.it (M.P.); alice.cola1@gmail.com (A.C.); m.barba8792@gmail.com (M.B.)

**Keywords:** prolapse, photobiostimulation, electrotherapy, stress urinary incontinence, women

## Abstract

**Introduction:** Pelvic floor disorders (PFDs) are a very widespread clinical phenomenon and are known to affect millions of women worldwide, significantly impairing their quality of life. New technologies and innovations such as electrotherapy and photobiostimulation are currently revolutionizing what concerns conservative treatment, allowing clinicians to tailor therapy to every woman. The aim of this study was to evaluate the feasibility of use of electrotherapy and photobiostimulation in PFD management and the initial results of treatment in clinical practice. **Methods:** This prospective study (IRB code: GSM-RF 2025) aimed to analyze the impact of the innovative DAFNE system to treat PFDs through electrotherapy and photobiostimulation. We enrolled patients with mild pelvic organ prolapse and stress urinary incontinence who desired a conservative treatment for their conditions. The following validated scales were used for assessing baseline quality of life before and after treatment: Urinary Distress Inventory-6 (UDI-6), Pelvic Organ Prolapse Distress Inventory-6 (POPDI-6), Female Sexual Function Index-6 (FSFI-6), 0–100 VAS Scale, and Vaginal Health Index. Electrotherapy was delivered through 4 gold rings, providing Functional Electrical Stimulation (FES), Transcutaneous Electrical Nerve Stimulation (TENS) and/or Microcurrent Electrical Neuromuscular Stimulation (MENS). The photobiostimulation system consisted of 24 LEDs per wavelength (420 nm UVA; 630 nm RED; 870 nm NIR/INFRARED) positioned at 360° along the entire length of the handpiece for antimicrobial purposes, microcirculation improvement, and anti-inflammatory action. The cycle of treatment consisted of 3–5 sessions, according to necessity. Overall improvement was measured through the Patient Global Impression of Improvement (PGI-I). Statistical analysis was performed using Student’s *t*-Test. A value of *p* < 0.05 was considered as significant. **Results:** In the period of interest, 32 women were treated with the DAFNE system. The mean age was 57.0 ± 16.6 years. Indications for treatment were stress urinary incontinence (16/32), mild pelvic organ prolapse (14/32), or both (2/32). The mean number of treatments per patient was 4. Quality of life improved as a consequence of the treatment according to the considered scales. Specifically, 87.5% of patients considered themselves improved. **Conclusions:** The DAFNE system has been successful in managing mild pelvic organ prolapse and stress urinary incontinence, improving quality of life scores. Embracing new technologies such as electrotherapy and photobiostimulation appears to be successful in conservatively managing a variety of PFDs.

## 1. Introduction

Pelvic floor disorders (PFDs) comprise a heterogeneous group of conditions arising from impaired structural support of the pelvic floor often manifesting as urinary, bowel, support, and sexual dysfunctions [1]. Obstetric trauma is widely recognized as the primary initiating event responsible for pelvic floor injury and for conferring long-term susceptibility to these disorders [2]. Nonetheless, biochemical and structural alterations within the connective tissue particularly involving collagen composition and enzymatic remodeling also play a critical role in pathogenesis [3], and several of these changes have been linked to the decline in estrogen associated with menopause [4]. Since pelvic floor disorders share multiple etiological factors, different conditions frequently coexist, recur, or develop as sequelae of therapeutic interventions, including reconstructive pelvic surgery [5,6]. For example, overactive bladder symptoms may improve following prolapse correction but can worsen when a suburethral sling is placed concomitantly [7]. Among PFDs, stress urinary incontinence and pelvic organ prolapse play a major role.

Stress urinary incontinence (SUI) represents one of the most prevalent and impactful pelvic floor disorders affecting nearly half of women in developed countries and carrying an estimated lifetime risk of surgical intervention of approximately 4% [8,9]. SUI is characterized by involuntary urine leakage during conditions where the intra-abdominal pressure exceeds urethral closure pressure, such as during coughing, exertion, or sneezing [10]. Pathophysiological mechanisms involve defects in periurethral connective tissue support leading to urethral hypermobility and intrinsic sphincter deficiency [11]. SUI may also occur de novo or persist following pelvic reconstructive procedures [12,13]. Its impacts on daily functioning include compromising social daily life, psychological well-being, and last but not least, sexual health [14,15]. In this kind of branch of medical condition, evaluation requires specialized expertise in order to explore concerns that patients possibly have difficulty dealing with and sharing with physicians, including sensitive domains related to quality of life and especially intimacy [16]. However, urodynamic testing can aid in differential diagnosis, considering that clinical and instrumental findings are often limited, and the utility of routine urodynamics remains debated due to variability in methodology and diagnostic criteria [17,18,19,20]. Current guidelines recommend conservative measures such as pelvic floor muscle training, behavioral modifications, and energy-based therapies as first-line treatment, reserving surgical options for women with persistent symptoms [21,22,23,24,25]. Among surgical interventions, midurethral slings represent the preferred approach due to their well-established safety and efficacy profile [26]. Retropubic slings, introduced in 1995, are widely considered the gold standard [27], while the transobturator route and subsequently single-incision slings were developed to minimize procedural risks associated with blind needle passage [24,28]. Nonetheless, all sling procedures may be associated with complications, including visceral injuries, chronic pelvic pain, voiding dysfunction, and de novo overactive bladder [29,30], supporting the prioritization of effective conservative alternatives when feasible [31]. Nevertheless, energy-based devices represent an effective non-invasive conservative therapy for SUI. Specifically, electromagnetic stimuli induce neuromuscular activation and strong pelvic floor contractions and muscular hypertrophy, which are related to significant improvements in symptom severity and quality of life [32,33,34].

Pelvic organ prolapse (POP), though, constitutes another common manifestation of pelvic floor dysfunction, arising from attenuation or failure of the connective and muscular support structures of the pelvic viscera [35]. It affects up to 50% of women with a history of at least one vaginal delivery, with incidence increasing markedly after menopause [35,36,37]. Risk factors include childbirth, aging, obesity, chronic increases in intra-abdominal pressure and underlying connective tissue abnormalities [38,39]. POP may be characterized by pelvic pressure, vaginal bulging, urinary or fecal dysfunction, incomplete emptying of the bladder and sexual impairment, significantly reducing quality of life and intimacy [40,41,42]. Prolapse surgery represents the keystone of treatment, especially for severe POP. However, management should be individualized with conservative treatment preferred in early stages to relieve symptoms and preserve pelvic function [43,44,45,46,47]. Pelvic floor muscle training remains the cornerstone of conservative care, although patients’ personal adherence and compliance challenges may limit its effectiveness [43,44,45,48]. Vaginal pessaries provide an alternative or complementary option, offering mechanical support and symptomatic relief for women who are not candidates for or prefer to postpone surgical treatment [36,37,43]. In this context, electromagnetic stimulation has emerged as a promising adjunctive conservative treatment for POP due to its ability to induce deep pelvic muscle contractions and muscular hypertrophy [49,50,51]. Moreover, it enhances neuromuscular activation and proprioceptive feedback, supporting, in this way, tissue regeneration, restoring neuromuscular function, modulating inflammatory pathways, and improving vaginal health parameters [52,53,54,55,56,57,58].

As Baruch et al. said [59], photobiomodulation (PBM) is a non-invasive energy-based therapy that uses, for medical purposes, a non-ionizing monochromatic light within the visible and near-infrared spectrum. PBM, in order to induce biological responses at the cellular level, relies on photochemical rather than thermal mechanisms. Low-dose lasers or light-emitting diodes (LEDs) are absorbed by endogenous chromophores, primarily mitochondrial cytochrome C oxidase, leading to an increase in mitochondrial activity and adenosine triphosphate (ATP) production [60,61,62,63,64,65].

These effects are associated with modulation of reactive oxygen species (ROS) and with the activation of redox-sensitive transcription factors involved in lots of biological mechanisms such as protein synthesis, collagen and elastin production, cell proliferation and tissue regeneration [66,67,68,69,70,71,72,73].

Different wavelengths exert specific biological effects. The red (620–700 nm) and near-infrared (700–1440 nm) wavelengths penetrate into deeper tissues and stimulate the activity of fibroblasts, enhancing extracellular matrix remodeling and promoting angiogenesis, thereby supporting tissue repair and neuromuscular recovery.

Conversely, blue light (400–500 nm) has more superficial activity and contributes to the modulation of cellular proliferation, wound healing and antimicrobial activity through ROS generation. A combined or sequential application of multiple wavelengths may provide synergistic effects and broaden the therapeutic impact of PBM.

Considering these mechanisms, PBM finds broad application in several clinical fields, also beyond the gynecologic field, including wound healing, scar remodeling, dermatological disorders, musculoskeletal conditions and chronic pain management.

In particular, in the field of urogynecology, PBM has shown potential benefits in the conservative management of pelvic floor disorders such as urinary incontinence, pelvic organ prolapse, dyspareunia and genitourinary syndrome of menopause.

Clinical studies have reported improvements in lots of areas of the quality of life of the patients: for example, in vaginal tissue quality, in lubrication and also in the management of pelvic pain, including dyspareunia symptoms, other than in urinary function [74,75,76,77,78,79].

PBM is generally considered safe, non-invasive, and drug-free. Although laser and LED devices differ in coherence, penetration depth, cost and treatment coverage, both technologies demonstrate favorable safety profiles. Clinical outcomes are dose-dependent and influenced by parameters such as wavelength, energy density, treatment duration, and session frequency. While standardized protocols are still lacking, low-intensity PBM devices represent a practical outpatient option for conservative pelvic floor management.

All these energy-based modalities are integrated within the DAFNE system which was developed to deliver personalized and multimodal therapeutic interventions perfectly tailored to each patient’s clinical diagnosis and needs. The DAFNE platform enables tailoring by the modulation of energy type, intensity, and treatment sequencing, allowing individualized treatment strategies [80,81,82,83].

The aim of our study was to evaluate the utility, feasibility, safety, and short-term clinical outcomes of electrical stimulation plus photobiostimulation, via DAFNE device, for the treatment of mild pelvic organ prolapse and stress urinary incontinence.

DAFNE devicewas provided by NOVAVISION Group based in Misinto (M), Italy.

## 2. Materials and Methods

This prospective study (IRB code: GSM-RF 2025) was carried out in a tertiary-level urogynecology department and included women with PFDs and aimed to analyze the impact of Functional Electrical Stimulation plus photobiostimulation to treat PFDs using electrotherapy and photobiostimulation.

This study was a pilot study structured as a prospective, single-center, clinical trial adopting a pre–post treatment comparative design. The primary endpoint was defined as how the clinical variation in validated patient-reported outcome measures, followed from baseline to the end of the therapeutic protocol of treatment. In addition, secondary endpoints included safety profile evaluation and patient-perceived global improvement.

Recruitment occurred from January 2025 to December 2025 during clinical gynecologic evaluations in Fondazione IRCCS San Gerardo dei Tintori, Monza, Italy.

The patients enrolled were those who desired conservative treatment for their clinical condition characterized by PFDs, including pelvic organ prolapse and stress urinary incontinence, and who chose management with energy-based devices. In total, the number of patients enrolled was 32, from 29 to 81 years old. Twenty-one patients were in their menopausal era, with no malignancy history or cardiologic/neurologic disorders or severe PFDs.

The eligibility criteria included women aged ≥18 years, with clinical diagnosis of mild pelvic organ prolapse (POP-Q stage I-II) and/or stress urinary incontinence—all confirmed by standardized urogynecologic examination with anamnesis, urodinamic exam and clinical evaluation—the persistence of symptoms for at least six months, and patients suitable for conservative management. The patients who were excluded were those who had contraindications according to manufacturers.

The POP-Q system, which is recognized as the international gold standard for the evaluation and staging of pelvic organ prolapse, was employed for the anatomical assessment.

The system quantifies descent at specific vaginal reference points (Aa, Ba, C, Ap, Bp, and D) in centimeters in comparison with the hymenal plane. The examinations were carried out by a single experienced urogynecologist at baseline and then at follow-up.

In particular, exclusion criteria comprised the following: pregnancy, active genitourinary infection, prior pelvic malignancy, presence of implantable electronic medical devices (e.g., cardiac pacemakers), severe pelvic organ prolapse (POP-Q stage III–IV), pelvic reconstructive surgery within the previous 12 months, neurological disorders affecting pelvic floor innervation, uncontrolled systemic disease, and any contraindication to electrotherapy or photobiomodulation. All participants provided written informed consent prior to enrollment. The study protocol was reviewed and approved by the local Institutional Review Board (ASST Monza; protocol code GSM-RF 2025, 27 December 2024) and was conducted in accordance with the Declaration of Helsinki.

At baseline, quality of life was measured by using lots of scales of evaluation such as the Urinary Distress Inventory-6 (UDI-6), the Pelvic Organ Prolapse Distress Inventory-6 (POPDI-6), the Female Sexual Function Index-6 (FSFI-6), the Vaginal Health Index (VHI) and a 0–100 VAS Scale indicating the total burden of pelvic floor symptoms.

All questionnaires were administered in a standardized manner by trained clinicians in order to strengthen methodological consistency.

Higher scores on UDI-6 and POPDI-6 scales correspond to greater symptom severity, whereas higher FSFI-6 scores reflect improved sexual function. The VHI was determined through objective clinical evaluation of vaginal elasticity, fluid volume, pH, epithelial integrity, and moisture.

A combination of Functional Electrical System (FES) and Photobiomodulation Therapy (PBMT) were delivered through the DAFNE system.

All procedures were performed in an outpatient setting by experienced urogynecologists specifically trained in device application, thereby ensuring procedural standardization, accuracy in technique, consistency and reproducibility.

**Figure 1:**
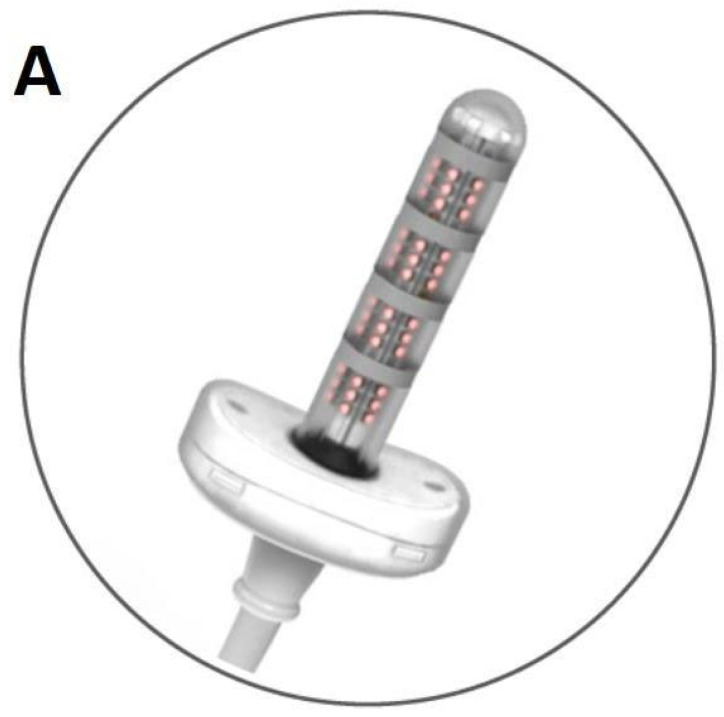
A: Electrotherapy plus photobiostimulation probe.

FES is delivered via an intravaginal probe through 4 gold rings, providing Functional Electrical Stimulation (FES), Transcutaneous Electrical Nerve Stimulation (TENS) and/or Microcurrent Electrical Neuromuscular Stimulator (MENS) (Figure 1).

The DAFNE system is an advanced, multimodal medical device developed for the treatment and functional restoration of gynecological conditions through a comprehensive bio-regenerative approach. By integrating photobiomodulation, electrical neuromodulation, electroporation and gas-assisted therapy within a single platform, the system is designed to answer to different pathological conditions including mucosal atrophy in the menopausal era or pelvic floor dysfunction. This integrated strategy enables simultaneous intervention on the cellular, vascular or neuromuscular level, promoting structural restoration and functional recovery using a controlled and minimally invasive treatment. Photobiomodulation (PBMT) works on mitochondrial stimulation and tissue remodeling and is the core of the DAFNE system, based on multi-wavelength LED emission. In fact, there is an enhancing effect of oxidative phosphorylation beyond adenosine triphosphate (ATP) synthesis. Increasing the availability of cellular energy, the fibroblast activation and collagen synthesis are promoted over the modulation of inflammatory mediators, thereby facilitating tissue repair and remodeling. The system incorporates distinct wavelengths, each associated with specific biological effects.

The photobiostimulation system consisted of 24 LEDs per wavelength (420 nm UVA; 630 nm RED; 870 nm NIR/INFRARED) positioned at 360° along the entire length of the handpiece in order to guarantee antimicrobial purpose, microcirculation improvement, and anti-inflammatory action. This was delivered through 48 LEDs (24 red, 24 infrared) equipped within the intravaginal probe. The radiant power of the device is approximately 1.728 W, delivering energy densities of 18–20 J/cm^2^ over 10 min sessions. The 420 nm wavelength (UV spectrum), though, was primarily employed for its antimicrobial properties and its modulation of superficial microbial load, whereas 630 nm (red spectrum) and 870 nm (near-infrared spectrum) were chosen for their well-known bio-stimulatory effects on angiogenesis, collagen synthesis, mitochondrial activation and modulation of inflammatory pathways. The 560 nm wavelength (green light spectrum) was chosen for the regulation of microvascular response and tissue homeostasis. The combined, multi-layer emission allows the stimulation of epithelial, stromal, and submucosal structures, optimizing tissue regeneration and functional recovery.

The Dafne platform integrates advanced electrical stimulation modalities to support neuromuscular re-education, pain modulation, and bio-regeneration, focusing on neuromodulation and pelvic floor rehabilitation. Delivered through safe, precisely controlled outputs with real-time monitoring, these modalities enhance both therapeutic efficacy and patient safety. The system includes TENS (Transcutaneous Electrical Nerve Stimulation) for the modulation of pain pathways and also for the management of the chronic pelvic pain; FES (Functional Electrical Stimulation), targeting the activation and strengthening of pelvic floor musculature; and MENS (Microcurrent Electrical Neuromuscular Stimulation), working on low-intensity cellular stimulation empowering tissue repair processes. This neuromodulatory component contributed to improved muscle tone, vascular perfusion and neuromuscular coordination. Electrical stimulation consisted of biphasic square wave pulses to activate and strengthen the pelvic floor muscles. Treatment parameters typically include a frequency range of 30–50 Hz for SUI, with a stimulation time of 20 min per session.

In addition, electroporation technology within the DAFNE system enables needle-free transmucosal delivery of bioactive substances. By transiently increasing cell membrane permeability, this technique facilitates the targeted penetration of therapeutic agents into tissues.

This approach enhances hydration and tissue regeneration, reducing the need for invasive methods, thereby improving patient comfort and compliance.

In conclusion, the DAFNE system incorporates controlled gas-assisted therapy using oxygen (O_2_) and carbon dioxide (CO_2_). This combined approach works on vasodilation, microcirculatory dynamics, and increases tissue oxygen availability, which plays a role in angiogenesis and collagen remodeling, also optimizing the metabolic environment needed for mucosal integrity and long-term tissue stability.

The intensity of stimulation was individually calibrated to achieve a targeted and customized therapy and to empower visible pelvic floor contraction without causing discomfort to the patient, thus optimizing neuromuscular recruitment while maintaining patient tolerability and compliance.

This multimodal, minimally invasive approach provides a grounded framework for restoring tissue vitality, improving pelvic floor function, and enhancing clinical outcomes in patients affected by gynecological pathologies.

Three to five sessions were delivered to each patient; sessions were scheduled at weekly intervals. The total number of sessions (the range was 3–5) was individualized according to baseline symptom severity and clinical response observed during the treatment.

After the treatment, the same scales were also used for determining quality of life after treatment. Overall improvement, though, was measured through the Patient Global Impression of Improvement (PGI-I). Any adverse effect was registered.

Adverse events were prospectively assessed at each visit, documented systematically and classified and graded according to severity (mild, moderate, severe) in order to determine the potential relationship and association to the intervention.

The anonymized data were entered into the database by the authors. Results were reported as mean ± standard deviation for continuous variables and as numbers (percentage) for non-continuous variables. Pre- and post-treatment data were compared to obtain objective and subjective results and tested for statistical significance. Differences were tested using a paired *t*-test for continuous data and Fisher’s exact test for non-continuous data. A *p* < 0.05 was considered as significant.

## 3. Results

During the study period, 32 women diagnosed with pelvic floor disorders (PFDs) underwent tailored combined treatment using the DAFNE system.

All enrolled participants successfully scheduled the full treatment protocol and post-treatment assessment, with no dropouts/losses to follow-up recorded during the study period.

The mean age of participants was 57.0 ± 16.6 years (Table 1), and a substantial proportion of participants were in their post-menopausal era.

Clinical indications included stress urinary incontinence (16/32 patients), mild pelvic organ prolapse (14/32 patients) or both (2/32 patients). Treatment was tailored to each patient’s clinical profile and needs, with an average of 3.8 sessions per patient (range 3–5).

Baseline quality of life scores and pelvic floor function outcomes are summarized in Table 2. With the combined electrical stimulation and photobiomodulation, the patient-reported outcomes in the scales demonstrate statistically and clinically significant improvements.

Specifically, Urinary Distress Inventory-6 (UDI-6) decreased from 24.0 ± 17.0 at baseline to 8.3 ± 10.0 post-treatment, corresponding to a mean reduction of −15.7 points. The standardized effect size was large (Cohen’s d = 1.13). This reduction exceeds the commonly reported minimal clinically important difference of approximately 11 points, indicating that the observed improvement is both statistically significant and clinically relevant. Moreover, in analyzing the Visual Analog Scale (VAS, 0–100) the global pelvic floor symptom burden decreased from 63.1 ± 13.6 to 28.4 ± 18.5 with a mean reduction of −34.7 points and a very large effect size (Cohen’s d = 2.14). Given that a reduction of 20 points is generally considered clinically meaningful, the observed change represents a substantial improvement in patients’ symptom severity.

Regarding Female Sexual Function Index-6 (FSFI-6), the scores increased from 8.6 ± 6.7 to 12.6 ± 8.6, corresponding to a mean improvement of +4.0 points (Cohen’s d = 0.52). This clinical evolution reflects a clinically perceptible enhancement in sexual function, which is a relevant point of consideration in the quality of life of a patient.

Additionally, Pelvic Organ Prolapse Distress Inventory-6 (POPDI-6) scores decreased from 17.1 ± 20.3 to 7.2 ± 11.5 with a mean reduction of −9.9 points and a moderate effect size (Cohen’s d = 0.60). Although these changes were statistically significant, they should be interpreted with caution in consideration of the multiple scales. In conclusion, Vaginal Health Index (VHI) scores showed a slight increase from 15.0 ± 4.6 to 15.5 ± 4.8, corresponding to a negligible effect size (Cohen’s d = 0.11). This change, actually, was neither statistically significant nor clinically meaningful, suggesting a slight short-term impact on vaginal trophism.

In consideration of the multiple outcomes, in order to address the risk of type I error, *p*-values were adjusted using the False Discovery Rate (Benjamini–Hochberg procedure). After correction, UDI-6 and VAS remained statistically significant (q < 0.01), confirming the strength of these primary outcomes. Improvements in FSFI-6 and POPDI-6, while significant before adjustment (*p* = 0.035 and *p* = 0.047, respectively), did not remain significant after FDR correction and should therefore be considered exploratory outcomes. VHI changes remained non-significant.

Overall, 87.5% of participants reported subjective improvement according to the Patient Global Impression of Improvement (PGI-I), with a mean score of 2.3 ± 0.9 further supporting the clinical importance of the treatment. No severe adverse events were observed.

**Figure 2:**
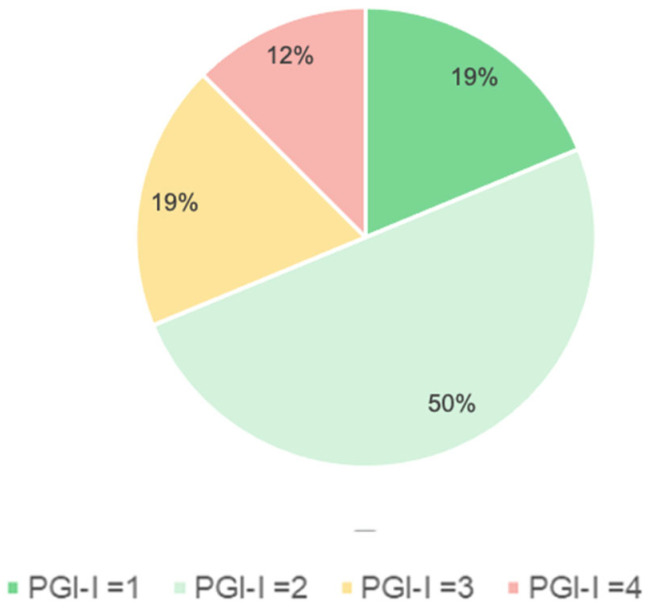
PG-I scores regarding improvements after treatment.

According to the Patient Global Impression of Improvement (PGI-I), 87.5% of participants reported perceived improvement, with a mean score of 2.3 ± 0.9 (Figure 2).

No severe adverse events were actually observed.

## 4. Discussion

This study aimed to underline the crucial role and the clinical efficacy of multimodal energy-based technologies in the conservative management of pelvic floor disorders (PFDs). Pelvic floor disorders represent a heterogeneous and multifactorial spectrum of clinical conditions characterized by structural connective tissue attenuation, neuromuscular dysfunction, impaired vascularization and chronic, low-grade inflammation. Consequently, therapeutic strategies targeting a single biological pathway may be considered inadequate to achieve a functional, structural and complete resolution of the clinical symptoms.

Both pelvic organ prolapse and stress urinary incontinence are conditions characterized by the weakening of pelvic floor muscles (PFM) and damage of the supporting structures. In this perspective, the combination of electrical stimulation (FES) and photobiostimulation (LED energy) may improve PFM tone, enhancing local microcirculation and promoting trophic and structural support to the pelvic organs.

Our study demonstrates that, concerning the conservative management of pelvic floor disorders (PFDs), it is essential to consider a multimodal energy-based approach. In fact, the combination of electrical stimulation (FES) and protobiostimulation (LED energy) could reveal itself to be much more effective than a single treatment by itself, both for the clinical outcome and for compliance, giving high importance to the satisfaction and especially the quality of life of the patients treated.

The DAFNE protocol—integrating electrotherapy and photobiomodulation—appears to yield therapeutic clinical and therapeutic effects through a combination of neuromodulatory, myofascial and bio-stimulatory mechanisms that target the multifactorial pathophysiology underlying mild pelvic organ prolapse and stress urinary incontinence [84,85,86,87].

On one hand, FES promotes the contraction of PFM, thus strengthening the muscle fibers and also improving proprioception and coordination during activities that are associated with an increase in intra-abdominal pressure. For this reason, improved PFM contraction capability reduces the hypermobility of the urethra and optimizes urethral closure pressures, decreasing in this way the frequency of urine leakage episodes.

On the other hand, though, red and near-red photobiostimulation is applied in the vagina using a probe in order to improve cellular metabolism, exerting a modulatory effect at mitochondrial level and promoting the circulation and the synthesis of collagen or local tissue repair. These physiological changes help restore and empower the support structure of the urethra, leading in this way to a decrease in involuntary leakage of urine. Talking about photobiostimulation (especially at 630 nm and 870 nm), it is important to underline its anti-inflammatory, analgesic and trophic effects on the vulvo-vaginal tissues and also on adjacent structures. Enhanced microcirculation and tissue regeneration appear to generate benefits in the pelvic floor environment, supporting both muscle training and long-term remodeling. Based on these combined mechanisms, the DAFNE system, on one side, aims to reduce SUI severity by improving voluntary pelvic contraction and, on the other side, enhance tissue support around the urethra [84,85,86].

Electrotherapy and photobiomodulation have been shown to highly influence cellular bioenergetics, mitochondrial activation, tissue oxygenation and neuromuscular transmission. In particular, photobiomodulation works on modulating cytochrome C oxidase-mediated ATP production, reducing oxidative stress and improving fibroblast activity, collagen remodeling and tissue elasticity at the end. All those factors are linked to pelvic connective tissue integrity. Electrotherapy, on one hand, plays a role in the recruitment of motor units, enhancing neuromuscular activation and working on neuroactivity of the pelvic floor. Concerning the conservative management of pelvic floor disorders, it is considered way over-superior in terms of efficacy tailoring the therapy with a multimodal therapeutic approach over a unimodal one [87,88,89,90].

The observed clinical benefits align with the literature supporting the role of energy-based therapies in pelvic floor rehabilitation. Studies investigating electrotherapy, radiofrequency and photobiomodulation have reported improvements in muscle tone regulation, tissue remodeling, and urogynecological symptom scores, though most evidence currently remains at the preliminary stage. The multimodal nature of the DAFNE system may represent an advanced way of tailored combined therapy over single-modality approaches by targeting, at the same time, the muscular and neural pathways beyond the connective-tissue components underlying PFDs.

This integrated approach is what could be considered as a tailored and more efficient way of management. Collectively, the present findings suggest that integrated energy-based treatment protocols such as DAFNE may represent a promising highly tailored treatment concerning conservative management for PFDs, providing a non-invasive approach capable of modulating both structural and neurofunctional components of pelvic support [87,88,89,90].

The findings of this study underline that the DAFNE multimodal energy-based system is a safe and effective conservative option for the management of mild pelvic organ prolapse and stress urinary incontinence. The favorable safety profile documented in this cohort of patients in this study underscores the feasibility and tolerability of multimodal energy-based protocols in appropriately selected patients looking for non-surgical therapeutic therapies.

Through the integrated use of electrotherapy and photobiomodulation, DAFNE appears to target multiple key domains that contribute to pelvic floor dysfunctions, promoting improvements in neuromuscular coordination, tissue trophism and overall pelvic support. The significant enhancement of patient-reported outcomes observed in this cohort suggests that what is working as a main factor is the synergic combination of all the treatments. In fact, bio-stimulation and neuromodulation may provide therapeutic benefits that exceed those associated with a single-modality conservative treatment. These results are consistent with the emerging literature highlighting the potential role of energy-based technologies in addition to conventional pelvic floor rehabilitation. Markedly, the favorable safety profile and high acceptability by patients observed during the study strengthen the rationale for broader implementation of this multimodal approach in appropriately selected patients [91,92,93,94,95,96].

In summary, the DAFNE system represents a promising, innovative and highly effective grounded option within the spectrum of conservative treatments for pelvic floor disorders. Its capacity to integrate neuromodulation and targeted biostimulation may contribute to reshaping current paradigms of non-invasive pelvic floor therapy.

Nevertheless, a limit of this study could be the relatively small sample size and the absence of a control group, which precludes broader generalizability. Larger, controlled prospective trials with longer follow-up are required to determine the durability of therapeutic effects and also to discriminate what the contribution is of each energetic modality, identifying the patient phenotypes that are most likely going to benefit from this combined therapeutic strategy. In addition, considering associated PFDs and symptoms, especially concerning genitourinary syndrome (GSM), 8–16% of women aged 18–64 experience vulvar pain or chronic vestibular pain, including chronic conditions such as lichen sclerosis: the goal for clinicians to customize the treatment is choosing multimodal platforms combining PBM and FES, adding electroporation when necessary in order to accelerate healing and restore tissues trophism [65,96].

However, the lack of a control group, the short follow-up and the updating of the patient-reported outcome measures just related to a short time could be considered important limitations of this study.

Future research should focus on defining all what concerns multimodal energetic therapy, in particular paying attention to protocols and optimal treatment parameters (frequency, intensity, duration, and sequencing of modalities) for different PFD phenotypes. Randomized controlled trials comparing DAFNE with standard pelvic floor rehabilitation and single-modality energy-based treatments will be crucial to validate the preliminary observations [65,95].

## 5. Conclusions

This study underlines that the DAFNE multimodal energy-based system represents a safe and effective conservative treatment for mild pelvic organ prolapse and stress urinary incontinence. By integrating electrotherapy and photobiomodulation, DAFNE targets multiple aspects of pelvic floor dysfunction, resulting in significant improvements for what concerns patient-reported outcomes. The DAFNE system represents a comprehensive, energy-based therapeutic platform for gynecological applications.

By integrating photonic stimulation, electrical neuromodulation, electroporation-mediated delivery and gas-assisted vascular enhancement, the device addresses key biological mechanisms involved in tissue degeneration and functional impairment, which are frequent conditions in women, especially during the menopausal era.

This approach addresses both functional and structural components of pelvic floor dysfunctions and was able to target a wide range of patients. The observed improvements in patient-reported outcomes suggest a synergistic effect of neuromodulation and biostimulation that is clinically relevant and that may exceed single-modality conservative approaches.

These preliminary findings align and contribute to the emerging evidence supporting the fact that energy-based technologies could be a part of a combined, multimodal and tailored therapy in the conservative management of pelvic floor disease (PFDs).

## Figures and Tables

**Table 1:** Population baseline characteristics. Continuous data are reported as mean (SD). Non-continuous data are reported as absolute (relative) frequency.

Age (years)	57.03 ± 16.57
Parity (n)	1.44 ± 0.91
Menopausal status	21 (65.6%)
Prolapse symptoms	16 (50%)
Stress urinary incontinence	20 (62.5%)

**Table 2:** Baseline and after-treatment quality of life scores. Data as mean ± standard deviation.

	Baseline	Post-Treatment	*p*-Value
POPDI-6	17.1 ± 20.3	7.2 ± 11.5	0.047
UDI-6	24.0 ± 17.0	8.3 ± 10.0	<0.001
0–100 VAS	63.1 ± 13.6	28.4 ± 18.5	<0.001
VHI	15.0 ± 4.6	15.5 ± 4.8	0.487
FSFI-6	8.6 ± 6.7	12.6 ± 8.6	0.035
PGI-I	n/A	2.3 ± 0.9	n/A

## Data Availability

The data that support the findings of this study are not openly available due to reasons of sensitivity and are available from the corresponding author upon reasonable request.
